# Female Body Dissatisfaction and Attentional Bias to Body Images Evaluated Using Visual Search

**DOI:** 10.3389/fpsyg.2019.02821

**Published:** 2020-01-22

**Authors:** John Cass, Georgina Giltrap, Daniel Talbot

**Affiliations:** ^1^School of Psychology, Western Sydney University, Sydney, NSW, Australia; ^2^School of Social Sciences and Psychology, Western Sydney University, Sydney, NSW, Australia

**Keywords:** visual search, attentional bias, body dissatisfaction, body image, body perception, body mass index

## Abstract

One factor, believed to predict body dissatisfaction is an individual’s propensity to attend to certain classes of human body image stimuli relative to other classes. These attentional biases have been evaluated using a range of paradigms, including dot-probe, eye-tracking and free view visual search, which have yielded a range of – often contradictory – findings. This study is the first to employ a classic compound visual search task to investigate the relationship between body dissatisfaction and attentional biases to images of underweight and with-overweight female bodies. Seventy-one undergraduate females, varying their degree of body dissatisfaction and Body Mass Index (BMI), searched for a horizontal or vertical target line among tilted lines. A separate female body image was presented within close proximity to each line. On average, faster search times were obtained when the target line was paired with a uniquely underweight or with-overweight body relative to neutral (average weight only) trials indicating that body weight-related images can effectively guide search. This *congruent* search effect was stronger for individuals with high eating restraint (a behavioral manifestation of body image disturbance) when search involved a uniquely underweight body. By contrast, individuals with high BMIs searched for lines more rapidly when paired with with-overweight rather than underweight bodies, than did individuals with lower BMIs. For *incongruent* trials – in which a unique body was paired with a distractor rather than the target – search times were indistinguishable from neutral trials, indicating that the deviant bodies neither compulsorily “captured” attention nor reduced participants’ ability to disengage their attention from either underweight or with-overweight bodies. These results imply the existence of attentional strategies which reflect one’s current body and goal-directed eating behaviors.

## Introduction

Body dissatisfaction refers to the negative subjective evaluation of one’s own physical shape, weight, and body (Stice and Shaw, 2002; Joseph et al., 2016). With high prevalence amongst women, it is considered a significant predictor of several major health risks, including depression, obesity, body dysmorphic disorder, anorexia nervosa, and bulimia nervosa (Kanayama et al., 2001; Keel and Klump, 2003; Hildebrandt et al., 2011; Mond et al., 2013; Fiske et al., 2014). Research indicates that individuals experiencing high levels of body dissatisfaction have cognitive biases, which influence the way they perceive themselves and their environment. Information processing related to body shape and size is presumed to occur automatically and with minimal conscious attention (Williamson et al., 2004). Numerous findings indicate that attentional biases to body-related stimuli are moderated by the degree to which an individual self-reports body dissatisfaction. The specific nature of attentional bias toward external body stimuli remains unclear, and is the focus of the present study.

In the domain of eating disorders, attentional biases favoring certain classes of food-related word and/or image stimuli have been proposed to reflect cognitive tendencies that serve to maintain particular pathological eating behaviors (Cooper et al., 1992; Faunce, 2002). The empirical relationships between attentional bias and eating disorder symptomology have been studied using a range of experimental methods. One of the most well-studied of these is the Stroop color naming task. Stroop studies show that individuals with significant eating disorder symptomology are often slower than controls to name the color of disorder-relevant stimuli (for example, a food-related word or with-overweight^1^ body image) than for the color-naming of disorder-neutral stimuli (Perpina et al., 1993; Walker et al., 1995; Sackville et al., 1998; Davidson and Wright, 2002).

These longer color naming latencies shown by subjects with high eating disorder symptomology are generally assumed to result from these subjects – either consciously or unconsciously – deploying a greater proportion of their limited attentional resources to the disorder-relevant stimulus compared to control subjects. It is unclear, however, whether this results from: (i) an attentional bias toward the disorder-relevant stimulus for those with high eating disorder symptoms (being more perceptually salient, for example); (ii) whether these subjects find it more difficult to disengage their attention from the disorder-relevant stimulus to the color naming task; or (iii) whether the disorder-relevant stimuli elicit a general degradation in performance for reasons possibly unrelated to attention.

Another task often used to investigate the relationship between attentional bias and eating disorder symptomology is the dot-probe task. The dot-probe paradigm involves the presentation of two stimuli (e.g., a thin-ideal body and a with-overweight anti-ideal body) displayed simultaneously for a brief duration (∼100–500 ms). Both of these images then disappear, one of them being replaced by a single target object (e.g., a line or an arrow) about which the participant is asked to make a simple perceptual judgment. Attentional bias is then calculated by comparing reaction times to judgments spatially associated with each class of body stimulus. A quicker reaction time to a target that replaces one type of stimulus (e.g., a with-overweight body) over another type (e.g., an underweight body) is interpreted as an attentional bias weighted toward the former stimulus.

Conceptually speaking, if the task also includes pairings with images that are neutral with respect to the dimension of interest (e.g., body fat), dot-probe tasks offer potential advantages over Stroop task in that they are able to disentangle attentional bias toward a particular class of stimulus from an ability to disengage attention from that stimulus (Jiang and Vartanian, 2018). Unfortunately, the results of these dot-probe studies are highly variable, conflicting and complex. Whereas some studies find associations between high body dissatisfaction and a reduced attentional bias toward underweight bodies (Glauert et al., 2010), others find apparently contradictory associations, with some associating body dissatisfaction with attentional biases toward images of bodies with-overweight (Glauert et al., 2010; Gao et al., 2013) and others attentional biases toward underweight body images (Cho and Lee, 2013; Joseph et al., 2016).

These apparent discrepancies may be accounted for by a range of methodological considerations. For example, taken as a whole, these studies employ a diverse array of body stimuli, including unclothed women (Glauert et al., 2010), clothed women with revealed torsos (Cho and Lee, 2013; Joseph et al., 2016), body parts varying in skin exposure, gesture and visual complexity (Gao et al., 2013, 2014). There is also considerable variation in the proportion of body fat represented in the images, particularly at the thin end of the spectrum (Glauert et al., 2010; Cho and Lee, 2013; Joseph et al., 2016). Such inconsistencies in the results of dot-probe experiments are not limited to the effects of body images. Indeed, Schmukle (2005) showed that dot-probe tasks offer very poor internal consistency and test–retest reliability. Eye-tracking studies have produced results broadly consistent with the idea that body dissatisfaction may be linked to some of the attentional biases described above, as evidenced through longer dwell times and more rapid fixation latencies toward both underweight and with-overweight body images relative to controls (Gao et al., 2013, 2014). In the absence of a clear and reliable relationship between the various measures of body dissatisfaction and attentional bias, however, we must consider other behavioral indices of attentional bias.

The other technique most commonly used by cognitive scientists to investigate attentional biases is visual search. In a standard visual search task observers are required to search for, and to make a perceptual judgment about, a uniquely defined target stimulus presented at some unknown location within an array of non-target (distractor) elements.

Few studies have applied the visual search paradigm to the question of attentional biases and body dissatisfaction (Jiang and Vartanian, 2018). Smeets et al. (2011) used an *odd-one-out* visual search task to examine attentional bias to body-related *words*, with body dissatisfaction induced through a body-checking priming manipulation. Those who had high body dissatisfaction showed speeded detection toward body-related words, compared to control conditions. Visual word stimuli, however, are known to recruit cortical mechanisms distinct from (but overlapping with) those involved in the processing of pictorial stimuli such as body stimuli (Downing et al., 2001; Taylor et al., 2007; Shinkareva et al., 2011; Park et al., 2014).

One study which did employ body images (Jiang and Vartanian, 2012) in conjunction with a visual search task, examined the extent to which participants’ eye fixations were drawn to task-irrelevant images of average weight bodies, underweight bodies, and bodies with overweight, whilst searching for a non-body target (a blue triangle). They found that fixational dwell times were longer when presented in the context of underweight and with-overweight body images than relative to non-body control images (signifying an attentional bias toward underweight and with-overweight bodies). No differences in dwell time were observed, however, for restrained and unrestrained eaters suggesting that visual search may be insensitive to differences in attentional biases in these two groups, should such differences exist. That said, the absence of such differences in attentional bias might be specific to the particular – and rather unconventional – *incidental fixation* measure used by Jiang and Vartanian (2012) to infer attentional bias. Whilst this incidental fixation task may be sensitive to the tendency of certain classes of body stimulus to involuntarily attract or capture subjects’ visual attention, what it does not assess is the extent to which subjects might voluntarily deploy their attention toward a certain body type to accomplish their search task.

**FIGURE 1:**
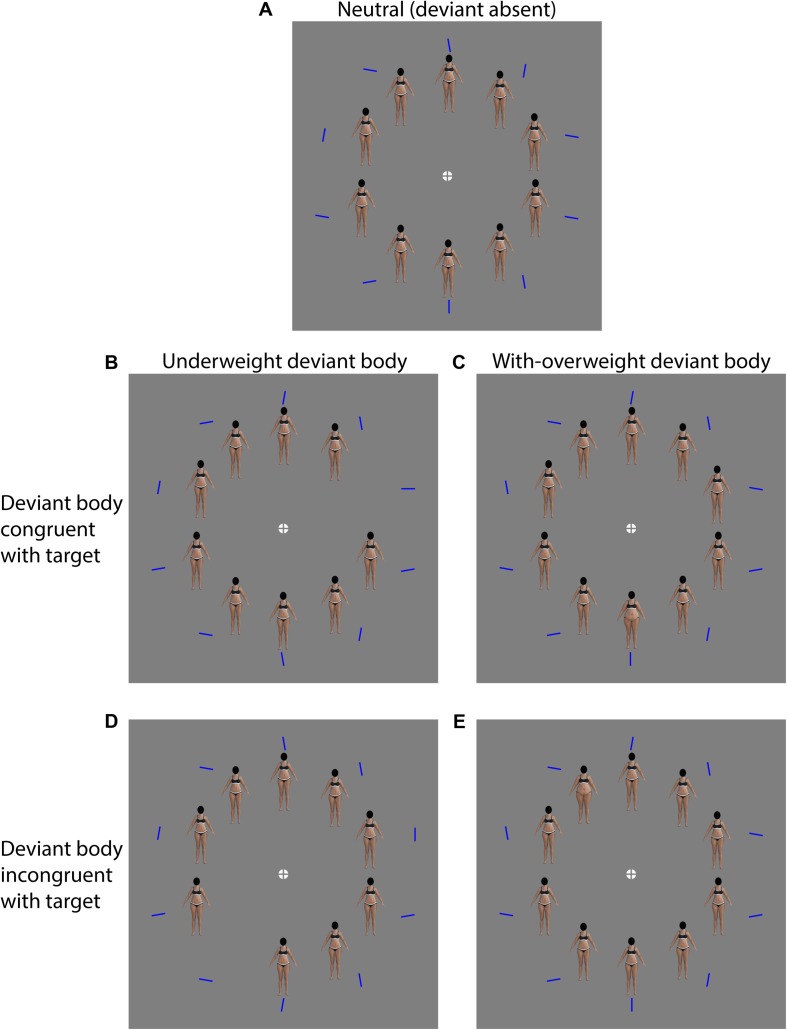
Examples of each experimental condition used in the visual search task. **(A)** Neutral condition: all bodies correspond to “normal weight” BMI. **(B)** Underweight deviant body is congruent with the target location. **(C)** With-overweight deviant body is congruent with the target location. **(D)** Underweight deviant body is congruent with one of the distractors (i.e., target incongruent). **(E)** With-overweight deviant body is congruent with one of the distractors (i.e., target incongruent).

The present experiment employs a *compound visual task* – a variant of the classic *odd-one-out* visual search paradigm – to investigate attentional biases to images of underweight, with-overweight and average-weight female bodies. A compound search task is composed of a primary and a (more theoretically interesting) secondary stimulus. In our case the *primary* stimulus is a single horizontal or vertical “target” line presented within an array of nine obliquely oriented distractors (±10 degrees from vertical or horizontal). Importantly, the location of the target line is randomized from trial to trial. The search task measures the accuracy and speed with which participants are able to identify the orientation of the target line. It is important to note that the primary orientation identification task is used only as a means of indexing search performance. That it involves the perceptual analysis of orientation is assumed to be unrelated to the processing of the *secondary* stimulus. The *secondary* stimulus used here involves an array of female body images, with each body image presented immediately adjacent to a particular *primary* line stimulus. On any given trial, all bodies on the screen are identical (neutral body types) (Figure 1A), except for a subset of trials in which one morphologically distinct body (referred to as the *deviant* body) is presented. Here the term *deviant* has no pejorative implication, referring only to the fact that a particular body image is different from the other bodies on the screen. Half of the deviant bodies in our study are images of underweight female bodies (Figures 1B,D), and the other half are with-overweight female bodies (Figures 1C,E) (see section “Methods,” for details). On the remaining trials (*neutral* trials), no underweight or with-overweight deviant bodies are presented. For *congruent* trials, an underweight or with-overweight deviant body is paired with the target line, with a neutral body paired with each distractor line (see Figures 1B,C). For *incongruent* trials the deviant body (underweight or with-overweight) is paired with a distractor line rather than the target line (see Figures 1D,E).

Attentional biases are inferred by comparing reaction times obtained on neutral trials to those obtained on congruent and incongruent trials. An advantage of the compound search paradigm over the conventional *odd-one-out* search paradigm is that it enables one to differentiate between attentional biases *toward* a given stimulus type (congruent effect) from subjects’ ability to disengage and shift attention *away* from stimuli which may have otherwise “captured” attention (incongruent effect).

Unlike the incidental fixation search paradigm employed by Jiang and Vartanian (2012), the compound search task offers the additional advantage of being able evaluate the existence of any bias that subjects might have in their ability and/or preference to deploy their goal-directed attention in the direction of a certain class of target *congruent* secondary stimulus – be it an underweight or a with-overweight body.

If female observers are able to use variations in the visual representation of human body fat/shape to guide their visual search, then we predict that search times will be faster on *congruent* trials, in which a deviant underweight or with-overweight body is uniquely paired with the target line (congruent trials), than on neutral trials. Moreover, if underweight or with-overweight body images compulsorily capture observers’ attention (i.e., without their conscious volition), such as is observed with “preattentive” visual features such as color or orientation singletons (Treisman and Gelade, 1980; Wolfe and Horowitz, 2004), we predict *search costs* on *incongruent* trials – i.e., slower search times on trials in which underweight or with-overweight bodies are uniquely paired with a distractor line compared to *neutral* trials in which no deviant body is presented.

In light of prior research demonstrating relationships between body dissatisfaction and attentional biases toward female body image-related stimuli (e.g., Glauert et al., 2010; Cho and Lee, 2013; Gao et al., 2013, 2014), this study also aims to: (i) establish whether attentional biases exist to both underweight and with-overweight body images; and (ii) determine whether the magnitudes of any such attentional biases in search performance are predicted by variations in female body dissatisfaction and/or BMI.

## Materials and Methods

### Participants

The sample included 71 students enrolled in undergraduate courses at Western Sydney University, aged between 18 and 46 years (mean = 21.63, *SD* = 5.04). Country of birth included Australia (80%), Hong Kong, Afghanistan, Kuwait, India, Vietnam, Greece, South Africa, China, Sudan, Pakistan, Nepal, Kenya, and Iran. No participants reported color blindness. Each participants’ height and weight were measured in order to calculate their BMI, which ranged from 16.21 to 46.29 kg/m^2^ (mean = 26.21, *SD* = 6.33). Participants either received course credit for their involvement in the research study or they were offered monetary compensation for their time. The Western Sydney University Human Ethics Committee approved the study (approval number H1778). Prior to the study, the experimental tasks and research procedure were explained to the participants. All participants provided written informed consent before initiating the study.

### Materials

#### Generation of the Figure Rating Scale

Fifteen computer generated female body images were designed and rendered using DAZStudio 4.9 Pro 3D modeling software to simulate BMIs ranging from 12.51 to 41.23 kg/m^2^. These body images were designed to simulate approximately equally spaced BMI categories ranging from emaciated (<15 kg/m^2^), underweight (15–18.5 kg/m^2^), through to normal (18.5–24.9 kg/m^2^), overweight (25.0–29.9 kg/m^2^), and obese (>30 kg/m^2^). Previous research indicates that facial features draw attention away from the body (Gardner et al., 2009) and attract the greatest proportion of eye fixations relative to other body regions (Warschburger et al., 2015), so the head of each body stimulus was occluded by a black ellipse. The bodies were rendered with black underwear, exposing full legs, torso, arms, and neck, areas shown to be important in the determination of body ideals (Crossley et al., 2012). Nudity was avoided as this has been shown to trigger specific attentional processing, similar to aversive stimuli (Most et al., 2007).

Two pilot studies were run using eight participants to select body images corresponding to the BMI subcategories depicted in the Photographic Figure Rating Scale (PFRS) (Swami et al., 2008, 2012). The PFRS has good construct validity, convergent validity and test–retest reliability, and includes photographs of actual female bodies representing a range of physically measured BMIs (Swami et al., 2008, 2012). A printed A2 sized paper version of the PFRS scale was produced using the images of the PFRS ranging from emaciated (1; extreme left) to obese (10; extreme right).

**FIGURE 2:**
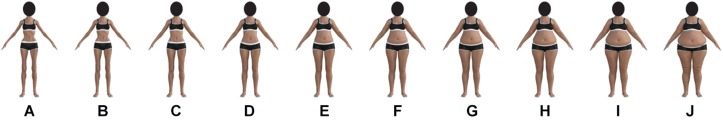
Computer-rendered female body stimuli used in the Figure Rating Scale. Labels **A–J** correspond to bodies with increasing BMI.

Each of the fifteen rendered female body stimuli were printed onto a separate card and combined into a single deck. For each participant, this deck was shuffled and handed to them with each card shown face down. Participants were instructed to turn over the top card within the deck and to examine the body image printed upon it. They were then asked to place this card face down onto the printed PFRS scale at a location corresponding to BMI depicted in the PFRS. This procedure was repeated until all fifteen body cards had been placed face down. At the completion of this procedure each of our rendered female body stimulus cards were ascribed a score based on their hypothetical position on the PFRS scale. For instance, if our thinnest rendered body image (ascribed “1”) was placed against the second-to leftmost body of the PFRS (category “2”), this received a difference score of 1. If the first body of the novel FRS was placed against the fourth category image of the PFRS, this received a difference score of 3. Scores each of our computer-generated body images were then averaged across participants. If the average difference score for a given rendered body image was less or greater than ±1 of a given PFRS subcategory, the stimuli were either discarded or remodeled and re-rendered until a complete set of rendered images were produced corresponding to each of the ten photographs shown in the PFRS. The second pilot study was run to confirm that the adjusted images more closely corresponded with the discrete visual body categories constituting the PFRS. These ten images were used in the main Figure Rating Scale experiment (Figure 2).

#### Administration of the FRS

The 10 rendered bodies selected for our FRS were presented on a computer screen as shown in Figure 2. Participants were asked to select the body most closely resembling their own body followed by the body most closely corresponding to their ideal body. Subjects registered their responses by typing the letter located beneath their selected body image on a computer keyboard. The overall FRS score was calculated by subtracting the number (where *a* = 1, *b* = 2, etc.) paired with the perceived ideal body from the number paired with the body representing the participant’s own body. The absolute value of this difference score was taken as an index of body dissatisfaction (Talbot et al., 2018a,b).

#### Eating Disorder Examination – Questionnaire

The Eating Disorder Examination Questionnaire (EDE-Q) (Fairburn and Beglin, 1994) was used to measure eating disorder symptomology for all participants. The EDE-Q is an adaptation of the Eating Disorder Examination, considered to be the gold standard for assessing the core features of anorexia nervosa, bulimia nervosa, and eating disorders not otherwise specified (Guest, 2000). The EDE-Q is widely used and has been shown to be a valid and reliable measure of eating disorders symptomology (Luce and Crowther, 1999; Grilo et al., 2001; Mond et al., 2004; Reas et al., 2006; Berg et al., 2012). It includes descriptive information regarding eating disorder psychopathology and is designed to assess specific behavioral symptoms such as excessive exercise, binge eating, diuretic misuse, laxative misuse and self-induced vomiting. It includes 28 questions, including 7-point, forced-choice rating schemes measured in terms of the number of days on which particular behaviors or attitudes occur. Participants receive a Global score, and a score for each of the four subscales relating to cognitive features of eating disorders. The four subscales include Restraint, Eating Concern, Shape Concern, Weight Concern.

#### Body Shape Questionnaire-34

The female-version of the Body Shape Questionnaire – 34 (Cooper et al., 1987) was administered to all participants. This is a self-report measure that assesses the levels of body dissatisfaction experienced during the past 4 weeks (28 days). This measure is the most commonly used measure in the literature on attentional bias and body dissatisfaction. Questions assess satisfaction and level of concern toward shape and weight, e.g., “Has being with thin women made you feel self-conscious about your shape?” and “Have you been so worried about your shape that you have been feeling you ought to diet?.” Participants are asked to rate how often they have experienced body/shape/weight related concerns from never to always. Participants receive a Global score of general body dissatisfaction, ranging from 34 to 206, with higher scores indicating higher levels of body dissatisfaction. The BSQ has been shown to be a valid and reliable measure of body dissatisfaction in women, α = 0.98 (Cooper et al., 1987; Varnado-Sullivan et al., 2006).

#### Body Mass Index

Body mass index was calculated for each participant by recording their body mass (in kg) and dividing it by the square of the height (cm) and expressed in units of kg/m^2^.

#### Visual Search Task

The visual search task was programed in MATLAB using the Psychtoolbox extensions (Brainard, 1997; Pelli, 1997; Kleiner et al., 2007). The stimulus was presented using a COMPAQ S920 cathode ray tube computer monitor. Screen resolution was set to 1024 × 768 pixels with a refresh rate of 85 Hz. Viewing distance was fixed at 340 mm. All images appeared against a background set to mid gray. At the beginning of each trial a gray fixation cross superimposed upon a white circle appeared at the center of the screen (see Figure 1). Then, 500 ms later, a primary stimulus and a secondary stimulus appeared. The primary stimulus was a single horizontal or vertical blue *target* line embedded with an array of nine oblique blue distractor lines (±10 degrees from vertical or horizontal). All lines were 0.7 mm in length, 1 mm in width. The line stimuli were regularly spaced on an invisible circle (radius = 13.5 cm) centered on the fixation cross. The location of the target line varied randomly from trial to trial so that the participant was unable to reliably predict its location.

Each primary target and distractor line stimulus was paired with a single female body image (the secondary stimulus). On average, each body image was 0.6 cm in height (top of “head” to soles of feet) and 0.3 cm in width (left to right finger tips). Each of these ten secondary body stimuli were positioned on a smaller invisible circle (radius = 9.5 cm) centered on the fixation cross. The center of each body image was located at the same polar angle (origin = fixation cross) as the center of its nearest primary line stimulus.

Across conditions, three different rendered bodies were presented: underweight-deviant body, a neutral body, and a with overweight body, representing three BMI categories: underweight (<18.5 kg/m^2^); normal (18.5–24.9 kg/m^2^); and obese (>30 kg/m^2^), respectively. This stimulus was a subset of images, rendered for the Figure Rating Scale. Again, these bodies were rendered with black underwear and their heads occluded by a black ellipse. For *congruent* trials, all bodies on the screen were identical average weight bodies, but for one deviant body (either underweight-deviant or with-overweight-deviant), which was paired with the target line. For *incongruent* trials, all bodies on the screen were identical average weight bodies but for one deviant body (either underweight-deviant or with overweight-deviant), which was paired with one of the distractor lines. For *neutral* trials, all bodies on the screen were identical average weight bodies with no underweight-deviant or overweight-deviant bodies presented. Examples of the main experimental conditions are depicted in Figure 1. Each participant was presented with 420 trials in total, including 224 congruent trials, 84 neutral trials and 112 incongruent trials. In half of the congruent and incongruent trials, an underweight-deviant body was displayed, and in the other half a with overweight-deviant body was displayed. There were more congruent trials than incongruent and neutral trials to encourage participants to use body shape as a cue to guide their search for the target line. Participants completed the task in two parts (210 trials each).

On each trial participants were instructed to identify the orientation of the target line as quickly and accurately as possible, using the left shift key for horizontal and the right shift key for vertical targets. Once the participant made a response, the primary and secondary stimulus disappeared, and the fixation point remained on the screen for 2 s between trials, to allow participants to center their gaze. The accuracy and speed with which participants were able to identify the orientation of the single unique target line was recorded.

### Procedure

Each participant was tested individually for approximately 1 h. After completion of the written consent form, participants completed (i) a demographic survey, (ii) the FRS, (iii) EDE-Q, and (iv) BSQ. These surveys and scales were all programed using Qualtrics software and administered via a desktop computer monitor and keyboard. Participants’ height and weight were recorded for BMI calculations. They were then seated in front of the computer screen at a viewing distance of 50 cm and received verbal instructions on the compound visual search task. Following this instruction each participant was presented with 15 practice trials. At the completion of the practice session, participants were able to ask questions if further clarity was required. Participants completed the subsequent visual search task in two blocks (approximately 18 min per block) with a 1–2 min break between each block.

## Results

Reaction time (RT) data from 71 participants were screened for accuracy, missing data, univariate and multivariate outliers. Data screening revealed no missing data and all recorded values were within a range appropriate to the variable scales. RTs for incorrect visual search trials (1358 trials) were omitted from the analyses (e.g., answered horizontal, when target was vertical, or vice versa). Additionally, RTs greater than 4000 ms were omitted from subsequent analysis to preclude task-irrelevant behavior, such as looking away from the screen, yielding on average 28.4% of trials per participant (S.E. = 1.3%) being omitted from subsequent analysis. Correct line judgments were then averaged across trial type, including (i) underweight-deviant congruent trials, (ii) with-overweight-deviant congruent trials, (iii) neutral trials, (iv) underweight-deviant incongruent trials, and (v) with-overweight-deviant congruent trials. BMI ranged from 16.21 to 46.29 (*M* = 26.21, *SD* = 6.33). BSQ ranged from 48 to 188 (*M* = 108.20, *SD* = 35.51). The average EDE-Q Global Score was 2.43 (*SD* = 1.45), which comprised the average of the four subscales, including, Restraint (*M* = 2.07, *SD* = 1.61), Eating Concern (*M* = 1.59, *SD* = 1.53), Shape Concern (*M* = 3.23, *SD* = 1.77), and Weight Concern (*M* = 2.81, *SD* = 1.58). Scores on the FRS, ranged from −8 to 6 (*M* = 2, *SD* = 2.07).

### Visual Search Effects

**FIGURE 3:**
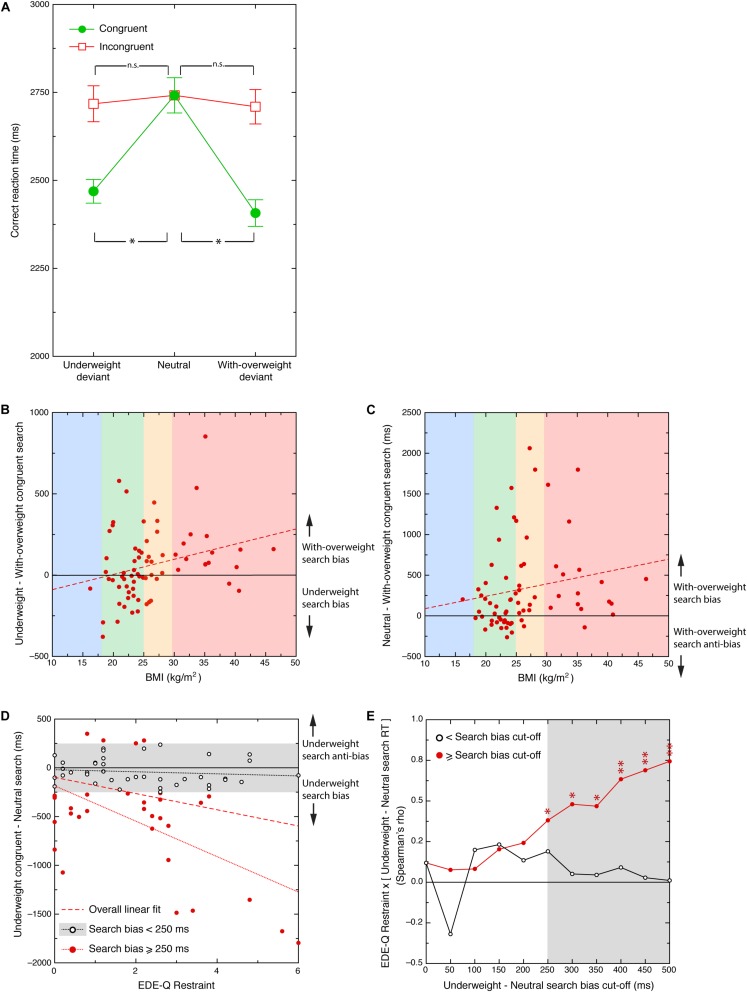
**(A)** Search times averaged across participants (*y*-axis) to congruent and incongruent trials (green circles and red squares, respectively) across the three body type conditions. Error bars are between-subject standard errors. Asterisks represent significant differences between both congruent deviant image conditions and the neutral (deviant absent) condition (*p* < 0.01). **(B)** Scatterplot showing the relationship between participant BMI and the difference in reaction time to underweight-deviant and with-overweight-deviant bodies on congruent trials. A positive difference score (*y*-axis) indicates faster search times to with-overweight relative to underweight body images (with-overweight search bias) and a negative difference score faster search to underweight relative to with-overweight body images (underweight search bias). The dashed line is the best fitting linear correlation. Vertically arranged colors represent conventional BMI categories. Unbroken horizontal line indicates zero bias. **(C)** Scatterplot of the relationship between participant BMI and the difference in reaction time on neutral and with-overweight-deviant congruent trials. Positive difference scores (*y*-axis) signify faster search times to with-overweight relative to neutral body images (with-overweight search bias). Negative difference scores indicate faster search to neutral relative to with-overweight body images (with-overweight search anti-bias). The dashed line is the best fitting linear correlation. Vertically arranged colors represent conventional BMI categories. Unbroken horizontal line indicates zero bias. **(D)** Scatterplot showing the relationship between EDE-Q Restraint and the difference in reaction time to underweight-congruent trials and neutral trials. A negative difference score indicates faster search to underweight relative to neutral body images (underweight bias) and a positive score, faster search to neutral relative to underweight body images (underweight anti-bias). Red dashed line is the overall best fitting linear correlation. White circles depict participants who produce weak (< ±250 ms) underweight search bias (within gray region), and red circles, participants with strong underweight search biases (≥ ±250 ms). Black and red dotted lines represent linear correlations between for these weak and strong search biased groups, respectively. Unbroken horizontal line indicates zero bias. **(E)** EDE-Q Restraint × [underweight – neutral] search time correlations across different levels of underweight-neutral search bias. Red circles/red line show participants with underweight search biases equal to above the variable bias cut-off, white circles/black line show participants with underweight search biases beneath the variable bias cut-off. Asterisks represent statistically significant Spearman’s rho values (^∗^*p* < 0.05; ^∗∗^*p* < 0.01). Gray region signifies underweight search bias, beyond which significant correlations with EDE-Q Restraint (±250 ms) are observed. Note, the Spearman’s rho value (*y*-axis) associated with a cut-off score of zero (*x*-axis) is equivalent to that of the red dashed linear fit in Figure 3D. Unbroken horizontal line indicates zero correlation.

The RTs for the congruent and incongruent trials for all participants across the three body size conditions (underweight, neutral and with-overweight), were analyzed using a 2 × 3 repeated measures analysis of variance (ANOVA). ANOVA test assumptions were satisfactory with the exception of Mauchly’s test of sphericity, which indicated that the sphericity assumption was not met for body type χ^2^(2) = 40.70, *p* < 0.001. Therefore, degrees of freedom were corrected for body type using the Greenhouse-Geisser estimates of sphericity (ε = 0.692). The main effect for congruency was significant, *F*(1, 70) = 27.51, *p* < 0.001. Means for congruent and incongruent trials were 2549 ms and 2721, respectively. The main effect for body size was also significant with a Greenhouse-Geisser adjustment to the degrees of freedom, *F*(1.38, 96.85) = 18.250, *p* < 0.001. Means for underweight body types, neutral body types and with overweight body types were 2593 ms, 2741 ms and 2559 ms, respectively. The congruency by body type trials interaction was significant with a Huynh-Feldt adjustment applied to the degrees of freedom (ε = 0.805), *F*(1.64, 114.92) = 21.07, *p* < 0.001 (see Figure 3A).

To deconstruct this interaction, five pairwise comparisons were conducted, using a Bonferroni adjusted alpha of 0.01 to adjust for family wise error. For congruent trials, average search times involving underweight-deviant bodies, *t*(70) = 5.02, *p* < 0.001, and those involving with-overweight-deviant bodies, *t*(70) = 5.24, *p* < 0.001, were both significantly faster than neutral trials in which no deviant underweight or with-overweight body was presented. Whilst a trend emerged indicating that for congruent trials, search times involving with-overweight-deviant bodies were often faster than those involving underweight-deviant bodies, this difference did not survive Bonferroni-adjustment, *t*(70) = 2.42, *p* = 0.09. For incongruent trials, there were no significant differences in average search times in the presence of either a with-overweight body, *t*(70) = −1.17, *p* = 0.245, or an underweight body, *t*(70) = −1.02, *p* = 0.313, when compared to neutral trials.

### Correlation Analyses

Scatterplots were examined to ensure linear and homoscedastic assumptions were met allowing for bivariate correlational analysis. Additionally, the scatterplots were screened for outliers. Upon inspection of the scatterplots of the correlation between the FRS and difference scores, one participant was identified as a significant outlier with a FRS score of −8. This outlier was removed from all subsequent analyses. Spearman product-moment correlations were performed between each measure of body dissatisfaction (BSQ, EDE-Q Global and subscales, FRS), BMI, and various visual search performance (RT) comparisons.

Significant linear correlations were found between the standard measures of body dissatisfaction (BSQ-34 and EDE-Q) and BMI (all *p*-values < 0.001). Our novel measure of body dissatisfaction, the FRS, correlated significantly with all other measures of body dissatisfaction as well as the BMI (all *p*-values < 0.001).

In order to examine linear associations between visual search performance (RTs) and our various psychological and physiological measures, a series of Spearman’s correlation analyses were conducted. Of the analyses involving raw average reaction times for each of *congruent*, *incongruent*, and *neutral* search conditions only one significant correlation was observed: a marginal positive linear association between neutral search times and BMI, *r_*s*_* = 0.236, *p* = 0.049. This suggests that for neutral trials, subjects with higher BMIs tended to take longer to complete the search task than do subjects with lower BMIs. This relationship, however, did not survive Bonferroni adjustment.

Additional correlational analyses were conducted based on a selection of RT difference scores. The first of these involved a measure of congruent underweight vs with-overweight search bias. This was calculated by subtracting *congruent with-overweight* search times from *congruent underweight* search times such that a positive value implies an overweight search bias, and a negative value, an underweight search bias. A significant correlation was observed between this measure of search bias and BMI, *r_*s*_* = 0.325, *p* = 0.006 (Bonferroni adjusted *p* = 0.048). Visual analysis of Figure 3B suggests that this positive association is principally driven by the predominantly with-overweight search biases exhibited by participants with BMIs > 25 kg/m^2^.

A full list of all correlational analyses performed can be found in Supplementary Table S1. To determine whether this with-overweight search bias was in fact driven principally driven by sensitivity to the with-overweight bodies, second of these RT difference score correlations involved a measure of with-overweight search bias that was not contingent upon performance in underweight conditions. This was calculated by subtracting *congruent with-overweight* search times from search times obtained in the *neutral* body condition, such that positive values imply that congruent trials containing with-overweight bodies produced faster search performance on average than neutral trials (with-overweight search bias), and negative values imply that overweight bodies slowed search relative to neutral trials (with-overweight search anti-bias). These difference scores exhibited a significant (non-adjusted) positive association with BMI, *r_*s*_* = 0.290, *p* = 0.015 (Figure 3C), indicating that higher BMI individuals tend to search for with-overweight targets more rapidly than they do on neutral trials, relative to lower BMI individuals.

Whilst significant correlations were observed between our measures of body dissatisfaction and (absolute or relative) search times. One interesting exception did emerge, however, in the relationship between EDE-Q Restraint and the difference in congruent search times involving underweight bodies compared to neutral conditions. This relative search variable is calculated by subtracting search times derived from neutral trials from those observed on congruent trials involving underweight bodies. According to this calculation, negative values correspond to faster search in trials involving underweight bodies, indicating an Underweight search bias, with positive values indicating an Underweight search anti-bias. Although there was no overall significant linear relationship between EDE-Q Restraint and neutral minus congruent underweight search times (red dashed line in Figure 3D), a highly significant linear relationship was evident once a critical level of underweight search bias was reached (±250 ms; red dotted line in Figure 3D). The effect of variation in this underweight bias cut-off on the linear association between EDE-Q Restraint and the magnitude of observed underweight search biases is shown in Figure 3E. In this figure, the strength of obtained Spearman’s rho correlations (*y*-axis) are expressed as a function various [underweight – neutral] bias cut-off values (*x*-axis). The figure shows significant linear dependencies between EDE-Q Restraint and underweight minus neutral search times for participants who produce (strong) underweight trial search biases of at least ±250 ms (red symbols) *r_*s*_* = 0.381, *p* = 0.016. Applying Bonferroni adjustments indexed by the number of underweight bias cut-offs compared in this analysis (11), reveals linear associations for underweight search biases of at least 400 ms (*r_*s*_* = 0.634, *p* = 0.022). By contrast, no linear association is observed for participants who produce (weak) underweight search biases, i.e., lower than any of the cut-offs tested (white circles).

## Discussion

This is the first study to systematically examine attentional bias to female body stimuli using a compound visual search paradigm. As hypothesized, results indicate that overall, participants performed the visual search task more rapidly when target lines were paired with a single underweight or with-overweight body image compared to neutral trials, which contained only average weight bodies. This demonstrates that variations in the visual representation of body fat (and lack thereof) can be used to guide visual search. Contrary to predictions, there were no significant differences in search latencies for incongruent trials relative to neutral trials. That pairing a uniquely underweight or with-overweight body image with a distractor line failed to produce significant search costs implies that the depicted variations in female body fat did not compulsorily capture our participants’ visual attention (Wolfe and Horowitz, 2004).

Contrary to our predictions, we found no evidence that high BMI is associated with faster search to underweight, idealized bodies relative to neutral (average weight only) body conditions. We did, however, observe a positive linear association between BMI and search times for underweight relative to with-overweight-congruent search conditions. Visual inspection of the scatterplot between these variables (Figure 3B) indicates that this association is predominantly driven by a with-overweight search bias combined with an almost complete absence of any thin search biases expressed by participants in the with-overweight BMI range (red region). By contrast, participants in the normal BMI range (green region) expressed a broad range of both underweight and with-overweight search biases. A similar trend is evident in Figure 3C, showing that most participants in higher BMI ranges express a with-overweight search bias relative to neutral conditions.

Both of these results imply that our higher range BMI participants tend to exhibit attentional biases favoring visual representations of human body mass that more closely resemble their own bodies. Whilst this might reflect the operation of complex social comparison processes, a more parsimonious explanation is that visual information that individuals are more familiar with tends to be processed more efficiently (and searched for more rapidly) than less familiar visual information (Qin et al., 2014). Assuming that our subjects are most visually familiar with their own bodies – as seen in the mirror, for example – it follows that body images more closely resembling our participants’ own bodies should elicit faster search than body images which are more dissimilar. Why this systematic visual search bias should be restricted to higher range BMI individuals is unknown. Given the diverse search biases expressed between individuals within the normal BMI range, it is conceivable that those in this range may be influenced by a more diverse set of factors, possibly involving historical experiences with one’s own body or the bodies of others.

On the face of it this tendency for our high BMI participants to exhibit an almost universal search bias favoring with-overweight bodies appears broadly inconsistent with a recent eye-movement study (Warschburger et al., 2015) showing that with-overweight individuals tend to produce more numerous and longer fixations to “more attractive” body regions than normal weight individuals. Given that with-overweight bodies tend to be rated as less attractive than normal weight bodies, if attractiveness were the sole determinant of attentional bias, based on the findings of Warschburger et al. (2015) we might expect with-overweight individuals to exhibit an underweight rather than a with-overweight search bias. Our results find the opposite relationship. It is worth noting that the line drawing body images used by Warschburger et al. (2015) did not vary in terms of their representation of body weight. Future research investigating the relative attractiveness of different body regions across different body shapes (e.g., underweight through to with-overweight) on both search and eye-movement behavior is necessary to determine what body-related factors drive attentional behavior in females.

The absence of any overall linear associations between visual search performance, the BSQ, EDE-Q and the FRS suggests that these psychological measures are not systematically related to our visual search task. A more nuanced analysis, however, reveals an interesting relationship between the EDE-Q Restraint subscale and the difference in search times on *congruent underweight* relative to *neutral* trials. By distinguishing between weakly and more strongly underweight search-biased subjects (ambivalent to the direction of this bias), we find that individuals who express a stronger search bias (≥ ±250 ms) exhibit a strong linear dependency between eating restraint and more rapid search for underweight relative to neutral bodies, with those scoring high on EDE-Q Restraint tending to benefit more from the presence of congruent underweight bodies than those low on EDE-Q Restraint. This suggests an attentional bias favoring underweight body types for individuals high in eating restraint. By contrast, those exhibiting a weak search bias did not show any such dependency. This dissociation between the strength (but not necessarily the direction) of underweight search bias suggests the existence of two sub-types in our population of female participants: (i) those whose attitudes toward eating behavior (restraint, in this case) are linked to their propensity to search for underweight relative to neutral bodies; and (ii) those whose attitudes toward eating are unrelated to their search behavior.

Whilst correlated to the other measures of body dissatisfaction, the eating restraint subscale of the EDE-Q is a behavioral manifestation of body image disturbance in that it centers on an individual’s desire to influence their weight and shape through certain active behaviors. Questions for this subscale center on strict dietary rules, limiting food intake, excluding foods from diet, desire for an empty stomach and restraining from eating for long periods of time. Restraint assesses specific behavioral symptoms that are active and goal-directed toward altering personal weight and shape (Penelo et al., 2013). A possible explanation for the attentional bias toward underweight bodies with high eating restraint (amongst search biased individuals) is that these individuals may engage in automatic upward social comparison processes (Rancourt et al., 2016). For instance, individuals high in restraint may look to thinner, ideal body types to gain information for self-improvement and to achieve their own body-image goals. Alternatively, this attentional bias may reflect a strategy which reinforces one’s goal-directed weight-restricted ideals and behaviors.

As expected, we observed strong positive correlations between participant BMI, and the various measures of body dissatisfaction and eating disorders (Hudson et al., 2007; Ro et al., 2012). Results also showed strong significant positive correlations between the FRS, and self-report measures of body shape dissatisfaction, eating disorder symptoms, and BMI. This suggests that our version of the FRS is a valid indicator of psychological and physiological variables related to body dissatisfaction. Given that figural rating scales offer numerous advantages over traditional self-report measures of body dissatisfaction (e.g., faster administration times, reduce interpretation biases, and non-reliant on language proficiency; Grogan, 2016), future research should seek to provide additional validity and test–retest reliability evidence for the FRS developed in the present study.

In terms of previous literature on the relationship between attentional bias to body shape and female body dissatisfaction the question remains as to how our results relate to the numerous and highly variable results derived from dot-probe, Stroop and eye-movement paradigms.

According to Jiang and Vartanian (2012), Stroop studies generally show greater interference for “fat-related” words such as “chubby” or “stomach” than for neutral words, particularly amongst restrained eaters. This general finding appears contrary to our findings which failed to observe any systematic relationship between eating restraint and attentional bias to with-overweight bodies. The strong linear correlation that we observe between eating restraint and search performance favoring underweight bodies (relative to average body weight conditions) suggests that eating restraint is better predicted by attentional bias to underweight than it is by attentional bias to with-overweight bodies. To our knowledge, no studies have compared the effects of thin- and fat-related words using a Stroop paradigm in a non-clinical population.

Studies that have sought to investigate the relationship between attentional bias to visual body weight information and body dissatisfaction using the more conventional dot-probe paradigm have reported a broad set of effects, ranging from no relationship with eating restraint (Boon et al., 2000; Smith and Rieger, 2010) to demonstration of a bias toward fat-related information and away from thin-related information in participants with eating disorders (Rieger et al., 1998; Shafran et al., 2007). Our observed relationship between restraint and attentional search biases favoring underweight bodies contradicts these findings. Why our paradigm should yield a different set of dependencies to those observed in the dot-probe paradigm remains an open question.

One possibility may lie in differences in volitional control. Whereas the dot probe involves very brief exposure durations (100–500 ms) and yields relatively short reaction times (typically 400–600 ms), the line and body stimuli used in our search task remained on the screen until the participant made a response, yielding much longer response times on average (∼2500–2700 ms). Both of these factors suggest that the attentional strategies participants use to perform each task may be very different. Whereas the dot-probe performance is likely to be informed by response strategies devoted to the appearance of dot-probe target elements which are not reliably linked a particular body type, in the case of our compound search task target line identification performance is likely to benefit from a more volitional analysis of the body image content (in congruent conditions, at least). Whilst it is tempting, therefore, to suggest that the different patterns of attentional bias observed in dot-probe and our compound search paradigm may reflect the operation of distinct strategic and/or attentional processes, the validity of this interpretation rests on future studies to compare the relative effects of different body types in each paradigm using identical sets of body stimuli.

Regarding our search stimuli, all body images used in our study were shown in identical poses from a single viewing perspective (frontoparallel plane) and all bodies had Caucasian skin tones. Both the viewpoint and surface characteristics of objects has been found to profoundly influence both brain responses and recognition performance across a variety of stimulus classes – including bodies (Chan et al., 2004; Favelle et al., 2011; Reppa et al., 2015). Ethnicity-linked visual cues have been found to differentially affect search behavior depending upon one’s own ethnicity (Zhou et al., 2015). Future research is required to determine the relative contribution of these various socio-cultural and imaged-based factors to both attentional biases to body weight information and body dissatisfaction.

With respect to our participants, given that pressures to meet certain body ideals are likely to differ greatly between age groups (Paxton et al., 1999; Wasylkiw and Williamson, 2013) it would be useful to examine a more broadly aged sample to better understand relationships between body ideals and attentional bias. Additionally, our study neglected muscularity – a facet of body image that is becoming increasingly important to women (Bozsik et al., 2018; Rodgers et al., 2018). Future studies should seek to examine the relationship between attention and female bodies that vary in terms of muscular tone and size. Another important factor to consider in future studies is the role of sex. Male body dissatisfaction, dysmorphia and disordered eating are prevalent yet relatively understudied (Cohane and Pope, 2001; Adams et al., 2005; Griffiths et al., 2014, 2018; Grogan, 2016). Although recent progress has been made (Griffiths et al., 2014; Smith et al., 2017; Talbot et al., 2018b) few studies have investigated the relationship between attentional bias and body dissatisfaction in the male population. A recent exception to this is a study by Talbot et al. (2019) which employed a similar compound search paradigm to that used here, but with male participants and images of male bodies. Interestingly, unlike the current study they found that measures of body dissatisfaction were associated with search performance involving images of with-overweight bodies. Why body dissatisfaction should be linked to search performance involving images of underweight bodies in females and images of with-overweight bodies in males is unknown, although it may point to strategic differences in the way females and males relate to body-weight related visual information.

## Data Availability Statement

The datasets generated for this study are available on request to the corresponding author.

## Ethics Statement

The studies involving human participants were reviewed and approved by the Western Sydney University Human Ethics Committee. The patients/participants provided their written informed consent to participate in this study.

## Author Contributions

JC designed the study, analyzed the data, and wrote the manuscript. GG ran the experiment, analyzed the data, and wrote the manuscript. DT wrote the manuscript.

## Conflict of Interest

The authors declare that the research was conducted in the absence of any commercial or financial relationships that could be construed as a potential conflict of interest.

## Footnotes

1 The term, ‘with-overweight’ is used throughout this manuscript at the suggestion of a reviewer.

## Supplementary Material

TABLE S1 Spearman correlations and associated uncorrected *p*-values between BMI and measures of body dissatisfaction.
