# Supplementary material for: Female Body Dissatisfaction and Attentional Bias to Body Images Evaluated Using Visual Search
Source: Front Psychol. 2020 Jan 22;10:2821. doi: 10.3389/fpsyg.2019.02821 (PMC6987376; doi:10.3389/fpsyg.2019.02821)
Supplement: TABLE S1 — Spearman correlations and associated uncorrected p-values between BMI and measures of body dissatisfaction. [file Data_Sheet_1.PDF]

Spearman Correlations

		BMI	BSQ-34 Score	EDEQ - Restraint	EDEQ - Eating Concern	EDEQ - Shape Concern	EDEQ - Weight Concern	EDEQ - Global Score	FRS Score
BMI	Spearman's rho	—							
	p-value	—							
BSQ-34 Score	Spearman's rho	0.401***	—						
	p-value	< .001	—						
EDEQ - Restraint	Spearman's rho	0.280*	0.573***	—					
	p-value	0.019	< .001	—					
EDEQ - Eating Concern	Spearman's rho	0.437***	0.655***	0.666***	—				
	p-value	< .001	< .001	< .001	—				
EDEQ - Shape Concern	Spearman's rho	0.425***	0.814***	0.647***	0.802***	—			
	p-value	< .001	< .001	< .001	< .001	—			
EDEQ - Weight Concern	Spearman's rho	0.550***	0.789***	0.682***	0.833***	0.923***	—		
	p-value	< .001	< .001	< .001	< .001	< .001	—		
EDEQ - Global Score	Spearman's rho	0.476***	0.781***	0.818***	0.891***	0.939***	0.950***	—	
	p-value	< .001	< .001	< .001	< .001	< .001	< .001	—	
FRS Score	Spearman's rho	0.627***	0.424***	0.361**	0.451***	0.569***	0.595***	0.552***	—
	p-value	< .001	< .001	0.002	< .001	< .001	< .001	< .001	—

* p < .05, ** p < .01, *** p < .001
